# Open Field Exercise Testing in Pediatric Congenital Heart Disease Patients: A Subsumption of Cardiovascular Parameters

**DOI:** 10.1007/s00246-023-03226-6

**Published:** 2023-07-24

**Authors:** J. Rückert, A. Michaelis, F. Markel, P. Kalden, F. Löffelbein, S. Klehs, I. Dähnert, I. Schöffl, K. Rottermann, C. Paech

**Affiliations:** 1 Department for Pediatric Cardiology, University of Leipzig - Heart Center, Strümpellstr. 39, 04289 Leipzig, Germany; 2 Department of Pediatric Cardiology, University Hospital Erlangen, Loschbergstraße 15, 91054 Erlangen, Germany

**Keywords:** Congenital heart disease, Children, CPET, Exercise testing

## Abstract

Heart failure is a common phenomenon in congenital heart disease patients. Cardiopulmonary exercise testing is used for a reliable assessment of heart failure but is still challenging, especially for young children. Implementing mobile cardiopulmonary exercise testing (CPET) can close that diagnostic gap. While average values for healthy children have already been published, this study aims to describe typical ranges of cardiovascular performance parameters of young children with congenital heart disease performing an 8-min running cardiopulmonary exercise test. Children aged 4–8 years with common congenital heart defects after corrective surgery (Tetralogy of Fallot; transposition of the great arteries and univentricular hearts after palliation) were included. The outdoor running protocol consisted of slow walking, slow jogging, fast jogging, and maximum speed running. Each exercise was performed for 2 min, except the last, in which children were instructed to keep up maximal speed as long as possible. A total of 78 children (45 male/33 female, mean age 6,24) with congenital heart disease participated in the study, of which 97% completed the CPET successfully. A detailed description of participating patients, including data on cardiac function and subjective fitness levels, is given to help physicians use this method to classify their patients. This study presents a typical range for cardiovascular performance parameters in a population of 4–8-year-old children with congenital heart disease tested in a newly developed outdoor running protocol for CPET.

## Introduction

Congenital heart disease patients often suffer from congestive heart failure or reduced physical exercise capacity [1]. Both situations are associated with a substantial morbidity and mortality in affected children [2]. While patients and parents easily recognize acute congestive heart failure due to new onset of symptoms, chronic congestive heart failure remains challenging to stratify as symptoms progress constantly and most patients are well adapted to their individual limits. It is crucial to catch the slow deterioration of physical exercise capacity in time to treat chronic congestive heart failure [3]. In addition, a reduced cardiovascular fitness, reflected by the lower $\dot{V}{O}_{2}peak$ values, represents an individual cardiovascular risk factor. In particular, a reduced $\dot{V}{O}_{2}peak$ at a young age seems to be hard to compensate throughout the adolescent and young adulthood period. This points to the importance of an early recognition of these parameters in young children with congenital heart disease, to provide the possibility for early intervention [4, 5].

Besides clinical examination, symptom-based classification systems [6, 7] and imaging techniques, such as MRI, CT and echocardiography, were performed. As a possible influence of baseline systolic or diastolic function may significantly influence the physical exercise capacity, echocardiographic parameters were taken to evaluate this possible confounder [8]. Furthermore, two main methods are used to grade heart failure in children. The first method is the measurement of brain natriuretic peptide (proBNP) levels [9, 10]. However, studies indicated that using proBNP as a prognostic value in adults cannot be equally applied to pediatric patients [7]. Moreover, venous blood sampling represents an invasive diagnostic tool and can be difficult and traumatizing for the child. The second method for a reliable assessment of congestive heart failure is cardiopulmonary exercise testing (CPET) [11]. This method is also considered the gold standard [12]. The peak oxygen uptake ($\dot{V}{O}_{2}peak$) [13] as well as the oxygen uptake efficiency slope (OUES) [14], a surrogate parameter for $\dot{V}{O}_{2}peak$, are important prognostic parameters in patients with congenital heart disease [15]. Typically, CPET has been performed on a bicycle or a treadmill [16] and it is a suitable diagnostic tool for adults and older children. However, specific reasons complicate these methods for use in young children. First, peak cardiopulmonary performance in children under the age of 8 years is difficult to achieve: on a bicycle because of weak leg muscle strength [17] and on a treadmill due to the unfamiliar exercise form and the fear to fall. Secondly, body size may limit the bicycle method [18].

The currently introduced method by Schöffl et al. [18] uses mobile cardiopulmonary exercise equipment with a protocol tailored to young children and closes that diagnostic gap. While average values for healthy children have already been established, this study evaluates a typical range of performance parameters in patients with congenital heart disease. Values are tested according to the reported protocol in terms of the individual anatomy and objective and subjective degree of congestive heart failure. The results should enable physicians to better assess the individual performance of children with congenital heart disease.

## Materials and Methods

During the period of 2021–2022, children aged 4–8 years with common congenital heart defects [Tetralogy of Fallot (TOF), Transposition of the great arteries (TGA), and after univentricular palliation (TCPC)] were included. These common, complex groups of CHD with symptomatic patients were selected based on clinical need according to the assessment of cardiovascular fitness and performance.

Voluntary participants were recruited consecutively with inclusion of children meeting the inclusion criteria. Written informed consent was obtained from each child and the respective parent using age-appropriate consent forms. This prospective investigator-initiated study was approved by the Ethics Committee of the University of Erlangen-Nuremberg (159_19B) and the Heart Center Leipzig (063/20-ek).

Before cardiopulmonary exercise testing, a thorough clinical examination was performed to rule out contraindications like an acute infection or other non-cardiac preexisting medical conditions. We obtained each patient’s history, an ECG, an echocardiography (including the baseline measurements for evaluation of systolic and diastolic cardiac function), a standardized questionnaire to evaluate subjective physical exercise capacity and a proBNP level. The mobile cardiopulmonary exercise testing device (Cortex Metamax 3B, Cortex Biophysik GmbH, Leipzig, Germany) was fitted to each child using a backpack that could be adapted accordingly to the respective size of each child. A respiratory mask for children (Hans Rudolph, Shawnee, Kansas, USA) was adapted using standard headgear. A Custo ECG 3-lead monitoring was installed.

All tests were performed on flat ground, either gravel or asphalt. A physician accompanied the children for instructions, motivation, and safety monitoring. The outdoor running protocol consisted of 2 min of slow walking, 2 min of slow jogging, 2 min of fast jogging, and a maximum of 2 min of maximum-speed running as previously described by Schöffl et al. [18]. Before each step, the children received instructions about the aimed speed and were then allowed to set the running speed for each exercise according to their capabilities. During the fastest stage, children were instructed to run as fast as possible for as long as possible and received verbal encouragement. The test ended with a recovery period of 3–5 min of slow walking.

A trained study nurse was at the exercise site to monitor the cardiopulmonary exercise testing recordings.

After test completion, values were analyzed using the Metasoft Studio software (Cortex Metamax 3B, Cortex Biophysik GmbH, Leipzig, Germany). Graphs were averaged over 20 data points. The following parameters were recorded constantly throughout the exercise test: oxygen uptake ($\dot{V}{O}_{2}$) as well as CO_2_ elimination ($\dot{VC}{O}_{2})$, heart rate, respiratory exchange ratio (RER = $\dot{\dot{V}{CO}_{2}/}$/$\dot{V}{O}_{2}/$), oxygen pulse (O_2_pulse = $\dot{V}{O}_{2}/$HR), minute ventilation ($\dot{V}E$) and the time of each test (exercise time). First ventilatory threshold (VT1) was calculated using the v-slope method [19] and second ventilatory threshold (VT2) was calculated according to Binder et al. [20]. Oxygen uptake efficiency slope was displayed as the slope of $\dot{V}{O}_{2}$ on the y-axis plotted against the logarithm of minute ventilation on the x-axis ($\dot{V}{O}_{2}$ a*log V ${}^{ \cdot }{\text{E}}$þb, a representing oxygen uptake efficiency slope in l/min). All OUES values were calculated from the onset of cardiopulmonary exercise testing to VT2 and were thus only reported in children having achieved VT2. Physiological criteria for having reached exhaustion included two criteria: peak HR ≥ 195/min, and/or RER at $\dot{V}{O}_{2}peak$ ≥ 1.0 [21].

Statistical analysis was calculated using SPSS V27 (IBM, Armonk, New York, USA). Comparisons of normally distributed CPET data between sexes were performed using two-sided T-test. For the comparison of the datasets for the patient characteristics, ANOVA testing was used. Differences in $V{O}_{2} peak$/OUES/kg values between TOF, TGA and TCPC patients were also compared using ANOVA testing. To evaluate the correlation between age and $V{O}_{2} peak$/OUES/kg, linear regression was performed. Pearson correlation coefficients were calculated to examine for possible associations between echocardiographic measurements and $V{O}_{2} peak$/OUES/kg values. Data are presented as mean values and were considered statistically significant with p < 0.05.

## Results

### Patient Characteristics

A total of 78 children with congenital heart disease performed CPET. Two children had to be excluded due to monitoring equipment failure (defect of heart rate sensor, data not stored and displayed in software) resulting in 97% technically flawless tests. 12 children were excluded from data analysis as they did not meet the exhausting criteria (peak HR ≥ 195/min, and/or RER at $\dot{V}{O}_{2}peak$ ≥ 1.0) because of patient imminent motivational reasons, that sometimes occur in young children. Of the remaining children, 64 completed the cardiopulmonary exercise test successfully while reaching their full exercise capacity.

Ranges of cardiovascular performance parameters are presented for a homogenous group of 24 TOF, 20 TGA, and 20 TCPC patients with no significant differences in baseline parameters like age, body weight, and systemic ventricular pump function. Only the echocardiographic parameters of tissue doppler, TAPSE and RV-Strain showed significant differences between the groups. All patients were Caucasian. Patient characteristics are shown in Table 1.

**Table 1:** Patient characteristics

	TOF	TGA	TCPC	p
Age (years)	5.96 ± 1.4 (4 to 8)	6.45 ± 1.3 (4 to 8)	6.85 ± 1.4 (4 to 8)	0.105
Weight (kg)	21.9 ± 6.7 (12.3 to 36.5)	24.8 ± 6.1 (13.4 to 35.4)	22.5 ± 6.1 (15.1 to 37.3)	0.304
Height (cm)	117.1 ± 11.6 (92.0 to 136.0)	122.8 ± 10.5 (97.0 to 137.0)	120.4 ± 12.1 (102.0 to 136.5)	0.269
BMI percentile	46.5 ± 31.2 (4 to 95)	48.0 ± 34.1 (2 to 99)	34.3 ± 32.0 (3 to 97)	0.342
BSA (m)	0.84 ± 0.17 (0.58 to 1.17)	0.92 ± 0.14 (0.60 to 1.11)	0.87 ± 0.16 (0.65 to 1.15)	0.267
Sex*	1.42	1.40	1.60	0.375
Male	14	12	8	
Female	10	8	12	
Total	24	20	20	
*1 = male				
2 = female				
LV-EF (%)	63.54 ± 6.78 (50 to 75)	66.11 ± 10.43 (50 to 89)	60.67 ± 11.18 (37 to 74)	0.280
GLPS (%)	− 17.73 ± 2.45 (− 21.7 to − 13.7)	− 18.77 ± 2.27 (− 23.0 to − 15.0)	− 18.34 ± 2.72 (− 21.0 to − 14.2)	0.575
RV-strain (%)	− 22.36 ± 3.83 (− 28.0 to − 14.0)	–	− 18.57 ± 3.95 (− 24 to − 15)	0.003*
Trans E	0.99 ± 0.21 (0.70 to 1.50)	0.93 ± 0.10 (0.70 to 1.0)	0.68 ± 0.21 (0.43 to 1.05)	< 0.001*
Trans A	0.37 ± 0.17 (0.18 to 0.80)	0.36 ± 0.09 (0.21 to 0.5)	0.40 ± 0.17 (0.15 to 0.71)	0.787
TAPSE (mm)	15.05 ± 3.74 (8 to 24)	14.90 ± 2.76 (11 to 23)	11.25 ± 4.13 (8 to 20)	0.029*
BNP (ng/l)	391.4 ± 488.0 (47.8 to 1240.0)	92.7 ± 61.4 (34.3 to 214.0)	144 ± 232.2 (23.7 to 615.0)	0.217
EF EBA*	1.09 ± 0.3 (1 to 2)	1.08 ± 0.28 (1 to 2)	1.29 ± 0.49 (1 to 2)	0.396
*1 = normal				
2 = moderate reduction				
Systemic ventricle				
Left	24	20	13	
Right	–	–	7	

Values are displayed as mean ± 1 standard deviation (minimum to maximum)

*Indicates p < 0.05

### CPET Data

Data from the cardiopulmonary exercise testing of children with Tetralogy of Fallot are presented in Table 2. There was a significant difference between girls and boys in O_2_ pulse (p = 0.033), HR at VT1 (p = 0.002) and $\dot{V}{O}_{2}$ at VT2 (p = 0.021).

**Table 2:** TOF

	Male	Female	p
VO_2_ peak (ml/(kg × min))	44.07 ± 6.46 (35–53)	42.80 ± 6.09 (35–55)	0.631
O_2_ puls	5.85 ± 1.21 (4–8)	4.70 ± 1.16 (3–6)	0.033*
RER at VO_2_ peak	1.13 ± 0.07 (1.02–1.23)	1.16 ± 0.06 (1.00–1.28)	0.272
VO_2_ at VT1 (l/min)	0.51 ± 0.16 (0.29–0.79)	0.47 ± 0.15 (0.28–0.66)	0.600
HR at VT1	115.8 ± 14.4 (98–149)	137.5 ± 12.7 (122–161)	0.002*
VO_2_ at VT2 (l/min)	0.79 ± 0.2 (0.44–1.17)	0.54 ± 0.13 (0.41–0.70)	0.021*
OUES (l/min)	1.10 ± 0.35 (0.40–1.50)	0.76 ± 0.24 (0.50–1.10)	0.063
Time of exercise (min)	7.62 ± 0.47 (6.66–8.00)	7.61 ± 0.47 (6.83–8.00)	0.989
OUES/kg	0.046 ± 0.011 (0.024–0.063)	0.043 ± 0.005 (0.036–0.049)	0.536

Values are displayed as mean ± 1 standard deviation (minimum/maximum)

*Indicates p < 0.05

Data from the cardiopulmonary exercise testing of children with Transposition of Great Arteries are presented in Table 3. $\dot{V}{O}_{2}$ at VT1 (p = 0.018) as well as OUES (l/min) (p < 0.01) and $\dot{V}{O}_{2}$ at VT2 (p < 0.01) are significantly different between the two sexes.

**Table 3:** TGA

	Male	Female	p
VO_2_ peak (ml/(kg × min))	46.67 ± 7.87 (38–62)	42.88 ± 7.02 (31–55)	0.286
O_2_ puls	6.75 ± 0.75 (6–8)	6.25 ± 2.77 (3–11)	0.632
RER at VO_2_ peak	1.11 ± 0.09 (1.00–1.30)	1.17 ± 0.08 (1.05–1.20)	0.156
VO_2_ at VT1 (l/min)	0.60 ± 0.09 (0.47–0.71)	0.47 ± 0.12 (0.30–0.62)	0.018*
HR at VT1	115.9 ± 10.3 (95–136)	117.2 ± 16.0 (88–129)	0.843
VO_2_ at VT2 (l/min)	0.98 ± 0.11 (0.85–1.25)	0.64 ± 0.12 (0.45–0.73)	< 0.001*
OUES (l/min)	1.35 ± 0.13 (1.00–1.50)	0.94 ± 0.19 (0.70–1.20)	< 0.001*
Time of exercise (min)	7.44 ± 0.81 (5.28–8.00)	7.80 ± 0.34 (7.00–8.00)	0.263
OUES/kg	0.051 ± 0.009 (0.035–0.067)	0.051 ± 0.007 (0.041–0.058)	0.984

Values are displayed as mean ± 1 standard deviation (minimum/maximum)

*Indicates p < 0.05

Data from the cardiopulmonary exercise testing of children after TCPC are presented in Table 4. There were no significant differences between girls and boys for any of the measured variables.

**Table 4:** TCPC

	Male	Female	p
VO_2_ peak (ml/(kg × min))	42.00 ± 4.93 (37–51)	37.00 ± 6.52 (27–47)	0.082
O_2_ puls	5.88 ± 1.36 (4–8)	4.92 ± 1.38 (3–7)	0.143
RER at VO_2_ peak	1.07 ± 0.07 (1.00–1.21)	1.11 ± 0.08 (1.03–1.21)	0.190
VO_2_ at VT1 (l/min)	0.48 ± 0.08 (0.39–0.61)	0.44 ± 0.13 (0.25–0.68)	0.430
HR at VT1	119.8 ± 26.0 (75–154)	120.3 ± 21.1 (76–156)	0.962
VO_2_ at VT2 (l/min)	0.76 ± 0.20 (0.54–1.09)	0.66 ± 0.26 (0.36–1.09)	0.414
OUES (l/min)	1.04 ± 0.29 (0.80–1.50)	0.93 ± 0.33 (0.50–1.40)	0.455
Time of exercise (min)	7.58 ± 0.74 (6.05–8.00)	7.32 ± 0.91 (5.33–8.00)	0.506
OUES/kg	0.045 ± 0.008 (0.030–0.053)	0.042 ± 0.008 (0.032–0.053)	0.341

Values are displayed as mean ± 1 standard deviation (minimum/maximum)

TGA patients reached the highest $V{O}_{2}peak$ values followed by patients with Tetralogy of Fallot and children with TCPC (Tables 2, 3, 4).

**Fig. 1:**
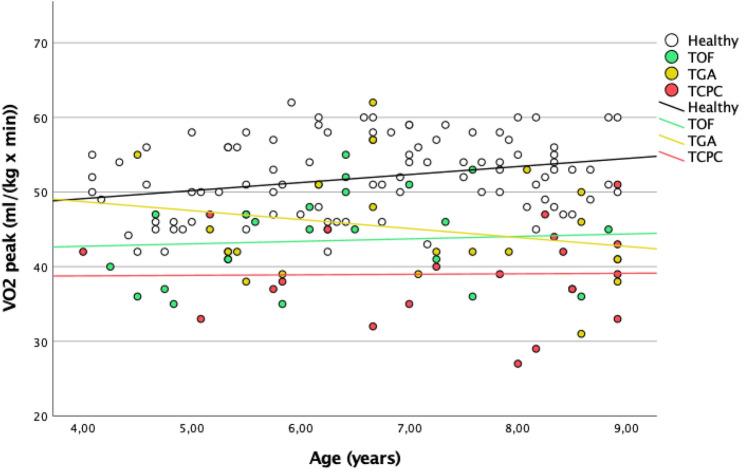
Correlation between Vo2 peak and age

**Fig. 2:**
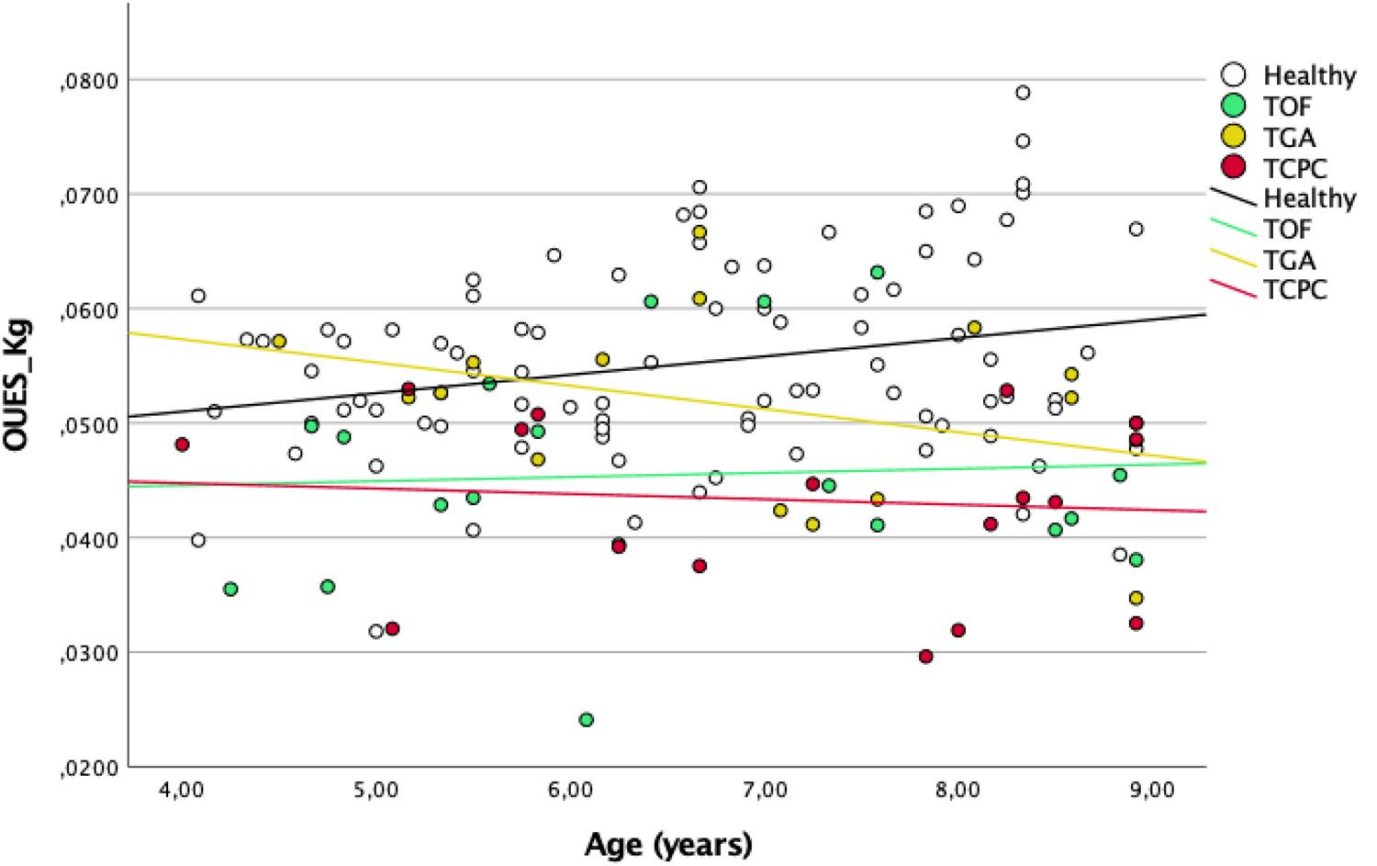
Correlation between Vo2 peak and OUES

Data from TOF, TGA, and TCPC patients were compared and showed a significant difference in $\dot{V}{O}_{2}peak$ (p = 0.014) and oxygen uptake efficiency (p = 0.026). The relationship between $\dot{V}{O}_{2}peak$, OUES and age in each congenital heart disease group using linear regression analysis is shown in Figs. 1 and 2.

Overall, there were no significant correlations between the $\dot{V}{O}_{2}peak$ and age [TOF to $\dot{V}{O}_{2}peak$, (p = 0.728); TGA to $\dot{V}{O}_{2}peak$ (p = 0.351); TCPC to $\dot{V}{O}_{2}peak$ (p = 0.945)].

The following formulas were deduced to determine a typical range of $\dot{V}{O}_{2}peak$/kg in children aged 4–8.

$${\text{TOF:}}\;\dot{V}O_{2} peak\left( {{{{\text{ml}}} \mathord{\left/ {\vphantom {{{\text{ml}}} {\left( {{\text{kg}}*{\text{minutes}}} \right)}}} \right. \kern-0pt} {\left( {{\text{kg}}*{\text{minutes}}} \right)}}} \right) = 41.41 + {\text{age}}\left( {{{{\text{months}}} \mathord{\left/ {\vphantom {{{\text{months}}} {12}}} \right. \kern-0pt} {12}}} \right)*0.33$$

$${\text{TGA:}}\;\dot{V}O_{2} peak\left( {{{{\text{ml}}} \mathord{\left/ {\vphantom {{{\text{ml}}} {\left( {{\text{kg}}*{\text{minutes}}} \right)}}} \right. \kern-0pt} {\left( {{\text{kg}}*{\text{minutes}}} \right)}}} \right) = 53.51{-}{\text{age}}\left( {{{{\text{months}}} \mathord{\left/ {\vphantom {{{\text{months}}} {12}}} \right. \kern-0pt} {12}}} \right)*1.2$$

$${\text{TCPC:}}\;\dot{V}O_{2} peak\left( {{{{\text{ml}}} \mathord{\left/ {\vphantom {{{\text{ml}}} {\left( {{\text{kg}}*{\text{minutes}}} \right)}}} \right. \kern-0pt} {\left( {{\text{kg}}*{\text{minutes}}} \right)}}} \right) = 38.49 + {\text{age}}\left( {{{{\text{months}}} \mathord{\left/ {\vphantom {{{\text{months}}} {12}}} \right. \kern-0pt} {12}}} \right)*0.07$$

Results of the correlation between OUES/kg and age groups are shown in Fig. 2. OUES/kg values did not differ significantly between the congenital heart disease groups with increasing age [TOF to OUES/kg (p = 0.823) TGA to OUES/kg (p = 0.178); TCPC to OUES/kg (p = 0.710)].

The following formulas were deduced to determine average values of OUES/kg in children aged 4–8.

$${\text{TOF: }}{{{\text{OUES}}} \mathord{\left/ {\vphantom {{{\text{OUES}}} {{\text{kg}}}}} \right. \kern-0pt} {{\text{kg}}}} = 0.045 = 0.04 + 3.61^{ - 4} *{\text{age}}\left( {{{{\text{month}}} \mathord{\left/ {\vphantom {{{\text{month}}} {12}}} \right. \kern-0pt} {12}}} \right)$$

$${\text{TGA: }}{{{\text{OUES}}} \mathord{\left/ {\vphantom {{{\text{OUES}}} {{\text{kg}}}}} \right. \kern-0pt} {{\text{kg}}}} = 0.051 = 0.07 - 2.03^{ - 3} *{\text{age }}\left( {{{{\text{month}}} \mathord{\left/ {\vphantom {{{\text{month}}} {12}}} \right. \kern-0pt} {12}}} \right)$$

$${\text{TCPC: }}{{{\text{OUES}}} \mathord{\left/ {\vphantom {{{\text{OUES}}} {{\text{kg}}}}} \right. \kern-0pt} {{\text{kg}}}} = 0.043 = 0.05 - 4.69^{ - 4} *{\text{age}}\left( {{{{\text{month}}} \mathord{\left/ {\vphantom {{{\text{month}}} {12}}} \right. \kern-0pt} {12}}} \right)$$

### Evaluation of Echocardiographic Parameters for the Prediction of ${\varvec{V}}{{\varvec{O}}}_{2}{\varvec{p}}{\varvec{e}}{\varvec{a}}{\varvec{k}}$

#### Influence of Cardiac Function on CPET Parameters

Data did not show a significant correlation between echocardiographic parameters and the oxygen uptake efficiency, LVEF, GLPS, RV-Strain, TAPSE, Trans-mitral E–A and $V{O}_{2}peak$ values (LVEF: p = 0.354; GLPS: p = 0.232; RV-Strain: 0.985).

## Discussion

The current study presents cardiovascular performance parameters for young children from 4 to 8 years with congenital heart disease, using an 8-min open-field cardiopulmonary exercise testing protocol. In recently published studies [18, 21], it has been shown that the 8-min open-field testing can be regarded as a new method capable of sufficiently performing cardiopulmonary exercise testing even when used in very young children. The presented data can underline the feasibility of the test for young children with congenital heart disease, as 97% of tests could be successfully completed.

As there are currently only reported values of the 8-min open-field-testing protocol for healthy children, the current data shall help physicians to interpret the cardiovascular performance of children with congenital heart disease.

Typical ranges of $\dot{V}{O}_{2}peak$ are depicted in Fig. 1 compared to the limits of healthy children. Overall, lower $V{O}_{2}peak$ values can be observed in congenital heart disease patients. Yet, a substantial number of patients with TGA or TOF seem to be able to reach normal $V{O}_{2}peak$ limits.

As shown in Tables 2, 3, and 4, it seems that TCPC patients show the lowest $V{O}_{2}peak$ values compared to patients with Tetralogy of Fallot and children with TGA. The statistical comparison between the individual groups can underline the evidence of our assumption (comparing TGA and TCPC: p = 0.008; TOF and TCPC: p = 0.021). With regard to other protocols for cardiopulmonary exercise testing, these findings are in accordance with the current literature [15]. With this in mind, the currently depicted 8-min open-field cardiopulmonary exercise testing seems to provide good quality data for CPET in young children with congenital heart disease.

Apart from the diagnosis, we identified no other influences on the $\dot{V}{O}_{2}peak$ values recorded in this study. In particular, there was no significant influence of cardiac function, evaluated by echocardiography, on CPET performance parameters. And in consistency with previous studies in healthy children, who, according to Kalden et al. [21], showed no significant differences of $\dot{V}{O}_{2}peak$ between healthy boys and girls in this range of age, the current data seem to underline these findings. We assume that differences in cardiopulmonary performance between sexes become visible only in older children and therefore, cannot be demonstrated in our study population of children of prepubertal age [16].

Consequently, the presented formulas to estimate an expectable $V{O}_{2} peak$/OUES/kg in a certain type of CHD are calculated independent of sex. While the type of congenital heart disease seems to predict a certain level of $V{O}_{2}peak$, the current study could not demonstrate any influence of echocardiographic parameters on $V{O}_{2}peak$. It is likely that several factors influence the $V{O}_{2}peak$. As a result, it cannot be expected that there is a significant influence of a single echocardiographic finding that might be found in a relatively small patient cohort.

It has to be stated that CPET measurements, like the $V{O}_{2}peak,$ differ with regard to the applied CPET protocol. For example, $V{O}_{2}peak$ is about 10% higher on a treadmill than on a cycle ergometer [12, 22]. Therefore it has to be kept in mind that the ranges reported in this study are linked to the reported CPET protocol. A comparison to other common protocols, with regard to maximum values, is difficult as mostly the percentage of really young children in these studies is limited [23, 24].

Finally, the study’s findings shall help physicians interpret the individual performance of young children with congenital heart disease and evaluate the patient for heart failure on the one hand side and to guide physical training to improve the cardiovascular risk profile in congenital heart disease patients on the other hand side.

## Conclusion

This study presents cardiovascular performance parameters of children aged 4–8 years with congenital heart disease tested in a newly developed outdoor running protocol for CPET.

## Limitations

In this study, only voluntary participants were included. Therefore, the possibility of a selection bias leading to an underrepresentation of children with high limitations of cardiopulmonary performance cannot be excluded. Almost exclusively Caucasian participants were included, limiting the applicability of results to children from other ethnic groups.
